# Changes in body composition and energetic efficiency in response to growth curve and dietary energy-to-protein ratio in broiler breeders

**DOI:** 10.1016/j.psj.2022.102410

**Published:** 2022-12-09

**Authors:** J. Heijmans, E. Beijer, M. Duijster, B. Kemp, R.P. Kwakkel, W.J.J. Gerrits, H. van den Brand

**Affiliations:** ⁎ De Heus Animal Nutrition B.V., 6717 VE Ede, the Netherlands; † Animal Nutrition Group, Department of Animal Sciences, Wageningen University, NL-6700 AH Wageningen, the Netherlands; ‡ Adaptation Physiology Group, Department of Animal Sciences, Wageningen University, NL-6700 AH Wageningen, the Netherlands

**Keywords:** body protein, body fat, lean tissue, adipose tissue, broiler breeder

## Abstract

Body composition plays an important role in reproduction in broiler breeders. The aim of this study was to evaluate the dynamics in body composition and energetic efficiency in broiler breeders, using different dietary strategies. About 1,536-day-old pullets were randomly allotted to 24 pens in a 2 × 4 factorial design with 2 growth curves (standard or elevated (+15%)) and 4 diets, with a step-wise increment in energy (96, 100, 104, and 108% apparent metabolizable energy nitrogen corrected [**AME_n_**]) fed on a pair-gain basis. Body composition was determined at 10 time points from 0 to 60 wk of age. Body protein mass was linearly related to body weight (**BW**) in growing breeders, which can be expressed as −6.4+0.184*BW (*R*^2^ = 0.99; *P* < 0.001). Body fat mass was exponentially related to BW in growing breeders, which can be expressed as $-42.2+50.8*1.0006^{BW}$ (*R*^2^ = 0.98; *P* < 0.001). A higher energy-to-protein ratio resulted in higher body fat mass at the same BW (*P* < 0.001). Sexual maturation was related to body protein mass at 21 wk of age, where each 100 g of body protein mass extra advanced sexual maturation by 5.4 d (*R*^2^ = 0.83). Estimates of energetic efficiency for growth (k_g_) and egg production (k_e_) appeared not constant, but varied with age in a quadratic manner between 0.27 and 0.54 for k_g_ and between 0.28 and 0.56 for k_e_. The quadratic relationship could be expressed as $k_{g}=\;0.408-0.0319*Age+0.00181*Age^{2}$ (*R*^2^ = 0.72; *P* < 0.001) and $k_{e}=-0.211+0.034*Age-0.00042*Age^{2}$ (*R*^2^ = 0.46; *P* < 0.001). Body protein mass in broiler breeders is tightly regulated and mainly depended on BW and seems to be the main determinant for sexual maturation. Body fat mass is exponentially related to BW, where an increase in dietary energy-to-protein ratio results in a higher body fat mass. Treatments had minimal effects on estimated energetic efficiencies in breeders.

## INTRODUCTION

Modern commercial broiler chickens are effective meat producers. They have been selected for decades for increased growth rate and high feed efficiency, leading to a high meat yield (Zuidhof et al., 2014). Broiler breeders hens, the mothers of broilers, also possess the genetics for a high growth rate. Growth rate and reproduction are negatively correlated and therefore broiler breeders are commonly fed restricted quantities of feed according to a targeted growth curve (**GC**) to prevent excessive weight gain and ensure reproductive success (Decuypere et al., 2010). This genetic selection has changed the body composition of broiler breeders over the last decades toward a higher lean mass and a lower fat mass (Eitan et al., 2014; Zuidhof et al., 2014).

Body reserves of a broiler breeder hen play an important role in reproduction. Several studies have emphasized the importance of the breeders’ metabolic status for sexual maturation (Bédécarrats et al., 2016; Hanlon et al., 2020; Van der Klein et al., 2020). Furthermore, it is suggested that body fat plays an important role in egg production (Van der Klein et al., 2018a), yolk synthesis (Salas et al., 2017), and laying persistency (Van Emous et al., 2015) and that body protein is an important source for albumen and yolk synthesis (Ekmay et al., 2014). Recently, concerns were raised that a biological limit in too low body fat mass for reproductive success may be approached or even reached in modern broiler breeder hens (Van der Klein et al., 2018a; Zuidhof, 2018; Hadinia et al., 2020). Changes in body composition might therefore influence reproductive success in broiler breeder hens. Development in body composition over different ages in broiler breeder hens has not been rigorously investigated before. Other studies have only considered body composition during the rearing period (Sakomura et al., 2003; De Los Mozos et al., 2017), during sexual maturation (Rabello et al., 2006; Hadinia et al., 2020), or during the production period (Caldas et al., 2018; Salas et al., 2019), only measured representatives of body composition, like abdominal fat pad and breast muscle weight (Van Emous et al., 2013; Lesuisse et al., 2017; Zuidhof, 2018), or only determined it at one specific age (Sun and Coon, 2005; Van der Klein et al., 2018a). The current study therefore aims to investigate body composition both during the rearing and production period in response to an altered GC and dietary energy-to-protein ratio. These 2 factors, have been shown to impact body composition in broiler breeder hens (Sun and Coon, 2005; Van Emous et al., 2013; De Los Mozos et al., 2017; Lesuisse et al., 2017; Van der Klein et al., 2018a; Salas et al., 2019).

Gaining insight in body composition development is also of importance for modeling energy partitioning in broiler breeders (Gous, 2015). In energy partitioning models, it is assumed that all dietary energy can be accounted for (Zuidhof, 2019). In a factorial approach, energy is partitioned into maintenance, growth of body protein, growth of body fat, and egg production (Sakomura, 2004; Zuidhof, 2019), the latter 3 are also referred to as retained energy. Body composition models in relation to dietary factors can help to determine the quantity of retained energy in breeder hens. The challenge in practice is to maximize energetic retention and minimize energy losses, which is also referred to as energetic efficiency. There have been attempts to quantify energetic efficiency in broiler breeders and the role of environmental factors in this energetic efficiency (Sakomura et al., 2003; Rabello et al., 2006; Reyes et al., 2011, 2012). It remains unclear whether or not dietary factors might affect energetic efficiency in broiler breeders. Quantifying dietary factors that contribute to energetic efficiency can help to design diets and feeding strategies to maximize energy retention. Furthermore, there are indications that energetic efficiency for body weight (**BW**) gain changes with age of the breeders (Sakomura et al., 2003), whereas most studies report a fixed value for energetic efficiency for BW gain or egg production, irrespective of age of the breeder (Rabello et al., 2006; Reyes et al., 2011, 2012).

The objective of the current study was to evaluate the development in body composition from pullet to mature broiler breeder hen, using different dietary strategies. Furthermore, we aimed to evaluate dynamics in energetic efficiency related to changes in body composition.

## MATERIALS AND METHODS

### Experimental Design

This experiment with female Ross 308 broiler breeders consisted of a 2 × 4 factorial arrangement with 2 GCs (standard growth curve [**SGC**] or elevated growth curve [**EGC**]) and 4 diets with different energy-to-protein ratio, created by a step-wise increase in apparent metabolizable energy nitrogen corrected (**AME_n_**; defined as 96, 100, 104, and 108% AME_n_ diet) at a similar CP content. Broiler breeders were allocated to the different treatments from hatch to 60 wk of age. Within each GC, feed allocation per diet was adapted weekly according to a paired-gain strategy. All experimental protocols were approved by the Central Commission on Animal Experimentation (the Hague, the Netherlands), approval number 2018.W-0023.001.

### Breeders, Housing and Management

Heijmans et al. (2021) reported a detailed description of this experiment. In short, at the start of the experiment (d 0), a total of 1,536 Ross 308 female broiler breeder day-old pullets were randomly assigned to 24 pens (64 pullets per pen) in 3 blocks of 8 pens (*n* = 3 per treatment). Each pen consisted of 2 areas: a floor area with wood shaving as bedding (4.9 m^2^) and an elevated slatted floor area (6.1 m^2^) with a track feeding system (9 m feeding length) with a grill to prevent rooster access to the feed, drinking nipples, perches (7.2 m) and laying nests. Until 20 wk of age, laying nests were covered with plastic to prevent access or sight to the laying nests. Breeders had ad libitum access to water. Pullets were kept at a photoperiod of 8L:16D (10 lux) until 21 wk of age. At 21 wk of age, pullets were photo-stimulated by an instant increase of the photoperiod to 11L:13D (20 lux), followed by a gradual increase to 13L:11D (40 lux) at 23 wk of age. At 20 wk of age, each pen was standardized to 45 breeders per pen closest to the average pen weight. At the same moment, 4 20-wk old Ross 308 roosters were placed per pen. A commercially available rooster diet (2,725 kcal of AME_n_/kg, 134 g of CP/kg, 5 g digestible lysine/kg) was provided to the roosters once per day in a rooster feeding pan. By adjusting the height of the feeding pan, female access to the rooster diet was prevented.

### Experimental Diets and Feed Allocation

Experimental diets were formulated with step-wise increment in dietary AME_n_ level from 96 to 108% AME_n_, where the 100% AME_n_ diet was according to breeder recommendations (Aviagen, 2016a). Diet was formulated isonitrogenous. A higher dietary AME_n_ level was realized by exchanging fibrous ingredients (cellulose and finely ground oat hulls) for energy rich ingredients (soy oil, lard, and maize starch), while maintaining a similar ratio between crude fat and starch. Table 1 presents the calculated and analyzed nutrient content of the 96% AME_n_ and 108% AME_n_ diets. The 100% AME_n_ and 104% AME_n_ diets were produced by mixing of the 96 and 108% AME_n_ diets in a 2:1 and 1:2 ratio, respectively. The experimental diets were provided ad libitum from day of placement until 2 wk of age. Hereafter, pens assigned to the SGC followed the breeder recommendation for BW (Aviagen, 2016b), whereas the EGC pens were fed to obtain a 15% higher BW throughout rearing and production. Daily feed allocation was calculated and adjusted weekly based on realized and desired growth per GC. Growth and egg production in the week prior were the directives for calculations of the daily feed allocation. Within each GC, daily feed allocation for each dietary energy-to-protein ratio was adapted according to a paired-gain strategy.

**Table 1:** Dietary ingredients, and calculated and analyzed nutrients of the 96% AME_n_ and 108% AME_n_ diets (g/kg, as-fed basis) of broiler breeders. The intermediate diets (100% AME_n_ and 104% AME_n_) were produced by mixing the 96% AME_n_ and 108% AME_n_ diets in a 2:1 (100% AME_n_) and 1:2 (104% AME_n_) ratio.

Item	Starter 1 (0–21 d)		Starter 2 (22–42 d)		Grower (43–112 d)		Prebreeder (113–160 d)		Breeder 1 (161–280 d)		Breeder 2 (281–420 d)	
Ingredient	96% AME_n_	108% AME_n_	96% AME_n_	108% AME_n_	96% AME_n_	108% AME_n_	96% AME_n_	108% AME_n_	96% AME_n_	108% AME_n_	96% AME_n_	108% AME_n_
Maize	450.0	450.0	500.0	500.0	400.0	400.0	500.0	500.0	440.0	440.0	460.0	460.0
Wheat	100.0	100.0	100.0	100.0	100.0	100.0	100.0	100.0	100.0	100.0	100.0	100.0
Soybean meal	240.9	245.1	141.3	146.3	76.1	80.7	48.9	52.8	149.8	152.5	130.5	133.4
Sunflower meal	50.0	50.0	90.0	90.0	150.0	150.0	165.0	165.0	80.0	80.0	90.0	90.0
Wheat middlings	-	-	-	-	100.0	100.0	25.0	25.0	-	-	-	-
Oat hulls (fine)	50.0	1.0	56.0	5.1	65.0	19.3	50.0	1.0	48.0	1.0	46.6	1.0
Cellulose	44.1	1.0	47.9	5.0	50.0	5.0	46.8	1.0	44.5	1.0	45.2	1.0
Soya oil	11.1	17.8	9.5	14.3	8.0	12.0	5.0	7.0	4.8	10.8	11.9	14.9
Lard	3.0	4.2	4.2	6.8	3.3	6.7	5.0	10.2	29.5	34.9	23.5	32.1
Maize starch	14.0	94.5	14.3	96.2	19.9	99.2	11.7	96.1	14.7	91.6	1.0	76.9
Limestone (fine)	13.9	14.1	13.8	13.9	13.3	13.4	-	-	-	-	-	-
Limestone (coarse)	-	-	-	-	-	-	24.5	24.6	71.0	71.1	73.4	73.5
Monocalcium phosphate	9.8	9.2	10.5	9.9	5.4	4.9	5.8	5.2	6.0	5.5	6.5	5.9
Sodium bicarbonate	3.3	3.3	3.3	3.3	2.5	2.5	3.3	3.3	2.7	2.7	3.0	2.9
Salt	1.8	1.8	1.7	1.7	2.2	2.2	1.5	1.5	2.1	2.1	2.0	2.0
L-Lysine	1.73	1.69	1.88	1.80	0.23	0.15	1.63	1.58	0.44	0.42	0.36	0.34
L-Threonine	0.68	0.68	0.54	0.54	-	-	0.49	0.48	0.57	0.58	0.54	0.55
DL-Methionine	2.34	2.34	1.71	1.71	0.65	0.65	1.13	1.13	1.73	1.77	1.59	1.62
Choline chloride 50%	0.8	0.8	0.8	0.8	0.8	0.8	1.5	1.4	1.4	1.3	1.5	1.4
Xylanase	0.1	0.1	0.1	0.1	0.1	0.1	0.1	0.1	0.1	0.1	0.1	0.1
Phytase	0.05	0.05	0.05	0.05	0.05	0.05	0.05	0.05	0.05	0.05	0.05	0.05
Premix rearing^1^	2.5	2.5	2.5	2.5	2.5	2.5	-	-	-	-	-	-
Premix laying^2^	-	-	-	-	-	-	2.5	2.5	2.5	2.5	2.5	2.5
Total	1000	1000	1000	1000	1000	1000	1000	1000	1000	1000	1000	1000
Calculated content^3^												
AME_n_ (kcal/kg)	2,570	2,890	2,570	2,890	2,545	2,865	2,640	2,970	2,735	3,080	2,735	3,080
Crude protein	175.1	175.0	143.7	143.6	136.5	136.5	123.0	122.5	138.5	137.7	135.2	134.3
Crude fat	41.5	49.0	42.0	49.0	40.0	47.0	38.8	45.7	60.0	71.1	61.6	72.8
Carbohydrates	535.6	569.0	558.1	592.1	546.4	580.5	557.4	593.6	507.3	538.1	502.9	535.8
Crude fiber	77.1	37.7	88.0	48.3	111.5	71.5	105.6	64.3	81.4	42.0	85.2	43.9
Starch	379.5	446.9	408.6	477.5	371.5	438.5	407.5	480.4	368.2	434.4	373.8	436.0
Starch:fat	9.1	9.1	9.7	9.7	9.3	9.3	10.5	10.5	6.1	6.1	6.1	6.0
Linoleic acid	18.0	21.0	18.0	20.3	17.0	19.0	16.3	17.4	16.8	20.0	20.0	22.0
Digestible lysine	9.0	9.0	7.0	7.0	4.8	4.8	5.1	5.1	5.9	5.9	5.5	5.5
Calcium	9.8	9.8	9.8	9.8	8.9	8.9	13.1	13.1	31.0	31.0	31.0	31.0
Retainable phosphorus	4.1	4.1	4.1	4.1	3.3	3.3	3.2	3.2	3.2	3.2	3.2	3.2
Analyzed content^4^												
Crude protein	170.2	172.9	145.1	148.0	133.0	135.1	129.6	127.4	145.2	142.2	139.9	135.1
Crude fat	37.0	43.2	38.3	44.3	39.0	42.4	33.1	41.1	57.6	66.8	58.3	68.7
Starch (Ewers)	401.0	463.0	408.0	472.0	377.0	431.0	415.6	486.3	376.4	436.8	371.7	432.5

1 Provided per kg diet: Vitamin A 10,000 IU; Vitamin D_3_ 3,000 IU; Vitamin E 100 IU; Vitamin K 3.0 mg; Vitamin B_1_ 3.0 mg; Vitamin B_2_ 6.0 mg; Vitamin B_6_ 4.0 mg; Vitamin B_12_ 20 μg; Niacinamide 35 mg; D-pantothenic acid 15 mg; Folic acid 1.5 mg; Biotin 0.20 mg; Iron 40 mg; Copper 16 mg; Manganese 120 mg; Zinc 90 mg; Iodine 1.25 mg; Selenium 0.3 mg.

2 Provided per kg diet: Vitamin A 10,000 IU; Vitamin D_3_ 3,000 IU; Vitamin E 100 IU; Vitamin K 5.0 mg; Vitamin B_1_ 3.0 mg; Vitamin B_2_ 12.0 mg; Vitamin B_6_ 5.0 mg; Vitamin B_12_ 40 μg; Niacinamide 55 mg; D-pantothenic acid 15 mg; Folic acid 2.0 mg; Biotin 0.40 mg; Iron 50 mg; Copper 10 mg; Manganese 120 mg; Zinc 90 mg; Iodine 2.0 mg; Selenium 0.3 mg.

3 Calculated according to CVB (2012).

4 Analysis according NEN-EN-ISO 16634-1 for crude protein, NEN-EN-ISO 6492-1999 for crude fat, and NEN-ISO 6493 for starch.

### Measurements

***Body weight.*** Body weight was measured weekly before feeding by individually weighing a minimum of 20 (rearing phase; 0–21 wk of age) or 15 (production phase; from 21 wk of age onward) randomly selected female breeders per pen. Every 3 (rearing phase) or 4 (production phase) wk all breeders within a pen were weighed.

***Egg production.*** Eggs were collected, graded (single or double yolked) and weighed daily per pen. Average egg weight was calculated per pen per week as the total egg weight, excluding weight of the double yolked eggs, divided by the number of single yolked eggs. Laying rate was calculated as the total number of eggs divided by the number of breeders per pen per week, corrected for mortality. Age at sexual maturity (**ASM**) was defined as age at 50% laying rate and was determined per pen by linear interpolation of age in days at which breeders passed 50% laying rate.

***Body composition.*** At d 0, 2-day-old pullets were selected for baseline measurement of body composition. Pullets were euthanized by a percussive blow to the head followed by cervical dislocation, weighed and pooled for body composition analysis. At 2, 6, 12, 16, 21, 28, 36, 46, and 60 wk of age, 2 female breeders per pen were selected before feeding within a range of approximately 2.5% of the average BW per treatment in that week. Selected breeders were euthanized by a percussive blow on the head followed by cervical dislocation and weighed (fresh BW). Breeders were then scalded for 30 s in water of approximately 65°C and defeathered by manual plucking. Breeders were then dissected and potential feed residues from the gastrointestinal tract were removed. From 12 wk of age onward, the abdominal fat pad, including fat surrounding the gizzard and proventriculus was removed, weighed and reinserted into the abdominal cavity. In case the oviduct contained egg components, these were removed as well, as these were not considered as part of the body composition. Hereafter, the defeathered carcass was weighed (feather-free BW). The defeathered carcass was ground to a homogeneous mixture of which a sample was analyzed for moisture, crude protein and crude fat content. Moisture content was determined by drying a sample at 103°C for 16 h (NEN-ISO-6496). Crude protein content was analyzed by the Kjeldahl method (NEN-ISO-8968-1). Crude fat content was analyzed by acid hydrolysis, using gravimetry (NEN-ISO-1735). Total body protein and body fat mass (g) were calculated respectively as crude protein or crude fat content multiplied with the feather-free BW in grams. At 2 wk of age, only 16 randomly selected pullets from the 2 extreme dietary treatments (96% AME_n_ and 108% AME_n_) were analyzed on body composition, because at that moment pullets were not yet feed restricted.

### Energy Efficiency Calculations

**Figure 1:**
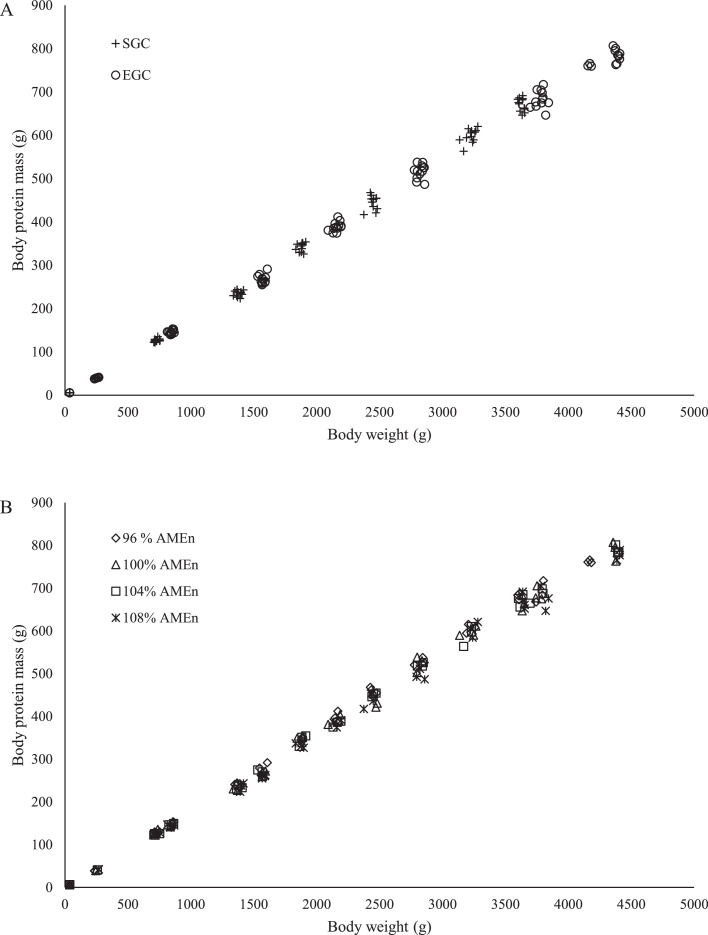
Relationship between body weight and body protein mass of broiler breeders between 0 and 36 wk of age fed at 2 different growth curves (A; SGC, standard growth curve or EGC, elevated growth curve (+15%); *n* = 12) and 4 diets (B), differing in energy-to-protein ratio (96, 100, 104, or 108% AME_n_; *n* = 6) from d 0 onward. Each symbol represents 1 replicate (pen) at each body weight.

**Figure 2:**
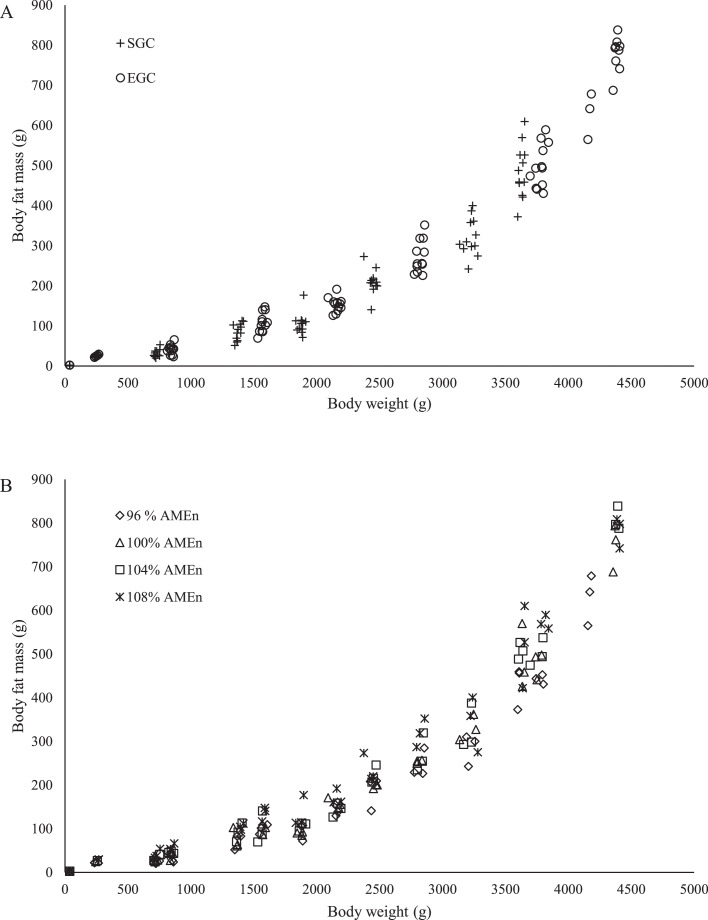
Relationship between body weight and body fat mass of a broiler breeder between 0 and 36 wk of age fed at 2 different growth curves (A; SGC, standard growth curve or EGC, elevated growth curve (+15%); *n* = 12) and 4 diets (B), differing in energy-to-protein ratio (96, 100, 104, or 108% AME_n_; *n* = 6) from d 0 onward. Each symbol represents 1 replicate (pen) at each body weight.

To calculate efficiency of energy utilization for BW gain (**k_g_**), data from the rearing phase was used in order to avoid bias in calculated values due to physiological processes involved in egg production. The following calculations were performed per pen per wk from 3 to 21 wk of age. Intake of AME_n_ (**ME_int_**) was calculated by multiplying feed intake with the dietary AME_n_ content. Metabolizable energy needed for maintenance (**ME_m_**) was calculated as 389 kcal * BP_m_^0.73^ * BP_t_/BP_m_ (Emmans, 1987), where BP_m_ is the mature body protein weight of 0.982 kg (calculated as ad libitum BW of 5.37 kg (Heck et al., 2004) times the body protein formula presented in the current study) and BP_t_ it the body protein weight in kilogram at timepoint *t*, which represents the degree of maturity in body protein. Body protein and body fat mass were predicted based on the formulas presented in the current study in relationship to BW (Figure 1, Figure 2). Body protein gain in grams (**BPG**) and body fat gain in grams (**BFG**) were calculated from initial (*t*) and final mass (*t* + 1). The energy retained as BW gain (**ER_g_**) was estimated by multiplying BPG and BFG by 5.4 and 9.3 kcal (Reyes et al., 2011), respectively, and then adding up these values. Metabolizable energy needed for BW gain (**ME_g_**) was calculated by dividing ER_g_ by k_g_. For calculation of k_g_, it was assumed that ME_int_ − ME_m_ − ME_g_ = 0. This leads to the following formula used for calculation of k_g_ per pen per week:

$$k_{g}=\frac{\left(5.4*BPG+9.3*BFG\right)}{\left(ME_{int}-\;ME_{m}\right)}$$

A 3 wk rolling average of k_g_ was used for further analysis. To calculate efficiency of energy utilization for egg production (**k_e_**) data from 36 to 60 wk of age was used in order to avoid bias in calculated values due to physiological processes involved in BW gain, as growth was minimized in this period (1 g/d on average). Average BW gain was calculated per pen and used for further calculations. In case average BW gain was negative, zero growth was assumed (3 pens) as it remains unclear whether or not a negative BW gain yields energy or if there is a cost factor involved as well. Similar calculations were used for ME_int_, ME_m_, BPG, BFG, and ER_g_ as described above. To calculate ME_g_ during the production period, average calculated k_g_ at 21 wk of age was used. Daily egg yolk and albumen production were estimated based on the formulas presented by Heijmans et al. (2022) multiplied with the daily egg mass production. Egg protein in grams (**EP**) and egg fat in grams (**EF**) mass were estimated by multiplying daily egg yolk and albumen mass in grams with the average crude protein and crude fat content in the yolk and albumen of Ross 308 breeders eggs (Nangsuay et al., 2015). The energy retained as egg (**ER_e_**) was estimated by multiplying EP and EF by 5.4 and 9.3 kcal (Reyes et al., 2011), respectively and then adding up these values. Metabolizable energy for egg production (**ME_e_**) was calculated by dividing ER_e_ by k_e_. For calculation of k_e_, it was assumed that ME_int_ − ME_m_ − ME_g_ − ME_e_ = 0. This leads to the following formula used for the calculation of k_e_ per pen per week:

$$k_{e}=\frac{\left(5.4*EP+9.3*EF\right)}{\left(ME_{int}-ME_{m}-ME_{g}\right)}$$

A 3 wk rolling average of k_e_ was used for further analysis.

### Statistical Analysis

Data on body composition were analyzed per time point, where pen was used as the experimental unit for all analyses. Data were analyzed using the restricted maximum likelihood variance components analysis procedure with a linear mixed model (Genstat 19th ed., 2019). The model used was:

(1) $$Y_{ijk}=\mu +GC_{i}+Diet_{j}+GC_{i}\;x\;Diet_{j}+Block_{k}+e_{ijk},$$

where *Y_ijk_* is the dependent variable, *µ* is the overall mean, GC*_i_* is the growth curve (*i* = SGC or EGC), Diet*_j_* is the energy-to-protein ratio in the diet (*j* = 96, 100, 104, or 108% AME_n_), GC*_i_* × Diet*_j_* is the interaction between GC and Diet, Block*_k_* is the block within the room (*k* = 1, 2, or 3), and *e_ijk_* is the residual error. Fisher adjustments were used for multiple comparisons of the factorial analysis. Additionally, effects of dietary energy-to-protein ratio were analyzed as linear or quadratic contrasts, also within GC. If linear effects were observed, the slope (*β*) is presented in the results section. If quadratic effects of dietary energy-to-protein ratio, also within GC, were observed, the estimated AME_n_ percentage at which the dependent variable was at the maximum (concave quadratic relation) or minimum (convex quadratic relation) was calculated and presented in the result section. Data are presented as LS means ± SEM.

In addition, linear and exponential regression curves were fitted in Genstat to describe body composition development in broiler breeders in relation to BW. Preliminary analysis showed no interaction between GC and dietary energy-to-protein ratio on body composition at each time point and therefore the regression curves are only presented on the main effects. Furthermore, preliminary analysis showed a high correlation between defeathered BW and fresh BW (*R*^2^ = 1.00) and therefore, for practical applicability of the presented formulas, fresh BW was used for further modeling. Preliminary analysis also showed a similar relationship between fresh BW and body composition in growing breeder pullets (0–21 wk of age) as in growing laying breeders (21–36 wk of age) and therefore body composition data were split into growing breeders (0–36 wk of age) and nongrowing, mature breeders (36–60 wk of age). For body protein mass in both growing and mature breeders and for body fat mass in mature breeders, preliminary analysis showed the highest *R*^2^ values and lowest Bayesian information criterion (**BIC**) for linear regression, compared to quadratic or exponential regression. A linear regression curve was therefore fitted, according to the following model:

(2) Y=a+b*BW,

where *Y* is either body protein mass in growing or mature breeders or body fat mass in mature breeders, a and b are the fitted coefficients for the linear regression curve and BW is the fresh BW of the breeder hen in grams. First, the model was fitted as single regression curve with the same coefficients for each GC or dietary energy-to-protein ratio (model I). Next, the model was step-wise expanded with a separate constant coefficient (a; model II) for parallel lines, or with a separate constant (a) plus linear (b; model III) coefficients for separate lines, for each GC × dietary energy-to-protein ratio interaction. After each model fit, it was evaluated whether or not the model significantly improved, compared to the previous model. Improvement was based on a significantly lower residual mean square error, a lower BIC, or a higher *R*^2^, compared to the previous model. The final model used (I to III) was the model that significantly improved the fit compared to the previous model, but no further significant improvement of the fit was observed of the next model.

For body fat mass in growing breeders only, preliminary analysis showed the highest *R*^2^ values and lowest BIC for exponential regression, compared to linear or quadratic regression. Therefore, an exponential regression curve was fitted for body fat mass in growing breeders:

(3) $$Y=a+b*c^{BW},$$

where *Y* is the body fat mass, a,b, and c are the fitted coefficients for the exponential regression curve and BW is the fresh BW of the breeder hen in grams. Similarly as model 2, a step-wise fitting and expansion was used as for each GC and/or dietary energy-to-protein ratio. The final model used, was the model that significantly improved the fit compared to the previous model, but no further significant improvement of the fit was observed of the next model.

Additionally, body protein mass and body fat mass were fitted against ASM in a multiple linear regression model:

(4) ASM=Bodyproteinmass(t)+Bodyfatmass(t),

where ASM is the age at sexual maturity (50% laying rate, in days), *t* represents the values at 6, 12, 16, or 21 wk of age. Body protein and body fat mass are expressed in grams.

Preliminary analysis showed the highest *R*^2^ values and lowest BIC for quadratic regression for dynamics of k_g_ and k_e_, compared to linear, linear-plateau or exponential regression. Therefore, for analysis of the dynamics of k_g_ and k_e_ a quadratic regression curve was fitted for each GC, dietary energy-to-protein ratio and GC × dietary energy-to-protein ratio:

(5) $$Y=a+b*Age+c*Age^{2},$$

where *Y* is the k_g_ or k_e_, a,b and c are the fitted coefficients for the quadratic regression curve and Age is the age of the breeder hen in wk. Similarly as model 2, a step-wise fitting and expansion was used as for each GC and/or dietary energy-to-protein ratio. The final model used was the model that significantly improved the fit compared to the previous model, but no further significant improvement of the fit was observed of the next model. Data are presented as LSmeans ± SEM. Estimated coefficients and *R*^2^ of fitted models are presented. Differences were reported where *P* ≤ 0.05.

## RESULTS

Results on nutrient intake, BW development, uniformity, productive performance, and egg composition are presented elsewhere (Heijmans et al., 2021, 2022).

### Body Composition

Defeathered BW of the selected breeders for body composition is presented in supplementary Table S1. Day-old breeder pullets had 5.9 g protein mass and 2.5 g fat mass in a body of 37.3 g. At 2 wk of age, pullets fed the 96% AME_n_ diet had a lower body protein (38.1 g) and fat (21.9 g) mass, compared to pullets fed the 108% AME_n_ diet (41.6 g and 29.4 g, respectively, *P* = 0.005 and *P* < 0.001). At none of the ages, an interaction was observed between breeder GC and dietary energy-to-protein ratio on body protein (Table 2) or fat mass (Table 3). At all ages, EGC breeders had a higher body protein and fat mass, compared to SGC breeders (*P* ≤ 0.02). Increasing dietary energy-to-protein ratio decreased body protein mass linearly at 12 (*β* = −0.9 g per % AME_n_), 16 (*β* = −1.3 g per % AME_n_), and 21 (*β* = −2.0 g per % AME_n_; *P* ≤ 0.05; Table 2) wk of age. At all other ages, no effect of dietary energy-to-protein was observed on body protein mass. Increasing dietary energy-to-protein ratio increased body fat mass linearly between 6 and 36 wk of age (*β* = 1.6, 2.8, 2.6, 5.1, 7.4, and 10.2 g per % AME_n_ at 6, 12, 16, 21, 28, and 36 wk of age, respectively; *P* ≤ 0.007; Table 3). At 46 wk of age, a quadratic effect was observed of dietary energy-to-protein ratio on body fat mass, where the lowest body fat mass was estimated at 102% AME_n_ (∆_max_ = 97.5 g; *P* = 0.04; Table 3). At 60 wk of age, no effect of dietary energy-to-protein ratio was observed on body fat mass (Table 3).

**Table 2:** Protein mass (g) in defeathered carcasses of broiler breeders from 6 to 60 wk of age fed at 2 different growth curves (SGC, standard growth curve or EGC, elevated growth curve (+15%)) and 4 diets, differing in energy-to-protein ratio (96, 100, 104, or 108% AME_n_) from 0 to 60 wk of age.

		Age (wk)							
Item		6	12	16	21	28	36	46	60
Growth curve (*n* = 12)									
SGC		126.5^b^	235.4^b^	342.5^b^	445.7^b^	598.9^b^	671.2^b^	680.9^b^	708.9^b^
EGC		146.2^a^	267.7^a^	389.2^a^	516.9^a^	683.6^a^	779.2^a^	758.2^a^	805.0^a^
SEM		1.3	2.5	2.7	3.7	5.6	4.5	5.7	7.8
Diet (*n* = 6)									
96% AME_n_		138.0	258.8	373.4^a^	494.5^a^	647.6	721.4	725.2	754.4
100% AME_n_		137.3	249.8	368.7^a^	479.3^ab^	641.2	726.2	732.4	764.2
104% AME_n_		135.8	250.7	364.0^ab^	485.3^a^	636.7	730.9	712.7	749.3
108% AME_n_		134.3	247.0	357.3^b^	466.0^b^	649.5	722.5	707.9	760.0
SEM		1.8	3.6	3.8	5.3	7.9	6.4	8.1	11.0
Treatment (*n* = 3)									
SGC	96% AME_n_	128.3	240.1	348.1	461.4	605.7	680.9	673.4	703.2
	100% AME_n_	128.3	233.4	345.8	435.3	596.7	663.9	699.6	707.3
	104% AME_n_	124.5	233.3	344.8	450.6	590.0	672.0	675.3	697.1
	108% AME_n_	124.9	234.9	331.2	435.4	603.4	668.0	675.3	728.3
EGC	96% AME_n_	147.7	277.5	398.7	527.7	689.5	761.9	777.1	805.7
	100% AME_n_	146.2	266.1	391.7	523.3	685.8	788.4	765.1	821.0
	104% AME_n_	147.1	268.1	383.1	520.0	683.3	789.7	750.1	801.6
	108% AME_n_	143.7	259.2	383.4	496.6	675.6	776.9	740.4	791.7
	SEM	2.5	5.1	5.3	7.5	11.2	9.0	11.4	15.5
*P* value									
Growth curve (GC)		<0.001	<0.001	<0.001	<0.001	<0.001	<0.001	<0.001	<0.001
Diet (factorial)		0.49	0.17	0.05	0.02	0.80	0.73	0.18	0.80
Diet (linear)		0.12	0.05	0.004	0.007	0.40	0.78	0.07	0.97
Diet (quadratic)		0.82	0.46	0.79	0.73	0.55	0.31	0.48	0.97
GC x Diet (factorial)		0.80	0.62	0.58	0.34	0.80	0.13	0.33	0.41
GC x Diet (linear)		0.86	0.26	0.93	0.53	0.66	0.19	0.17	0.21
GC x Diet (quadratic)		0.74	0.69	0.22	0.22	0.39	0.05	0.40	0.24

ab LSmeans within a column and factor lacking a common superscript differ (*P* ≤ 0.05).

**Table 3:** Fat mass (g) in defeathered carcasses of broiler breeders from 6 to 60 wk of age fed at 2 different growth curves (SGC, standard growth curve or EGC, elevated growth curve (+15%)) and 4 diets, differing in energy-to-protein ratio (96, 100, 104, or 108% AME_n_) from 0 to 60 wk of age.

		Age (wk)							
Item		6	12	16	21	28	36	46	60
Growth curve (*n* = 12)									
SGC		31.7^b^	85.9^b^	106.2^b^	210.4^b^	321.3^b^	485.2^b^	415.8^b^	531.9^b^
EGC		41.4^a^	108.2^a^	153.5^a^	272.1^a^	498.3^a^	741.5^a^	706.4^a^	670.8^a^
SEM		2.5	5.8	5.3	9.1	10.3	17.4	12.8	35.8
Diet (*n* = 6)									
96% AME_n_		27.1^c^	85.6^b^	118.1^b^	216.2^b^	363.2^c^	529.0^b^	605.0^a^	636.6
100% AME_n_		33.8^bc^	87.5^b^	123.6^b^	225.5^b^	404.0^bc^	616.1^a^	507.5^b^	534.7
104% AME_n_		38.4^ab^	95.3^ab^	124.7^b^	246.0^ab^	414.0^ab^	657.3^a^	564.4^a^	616.2
108% AME_n_		46.9^a^	119.7^a^	153.0^a^	277.3^a^	458.1^a^	651.0^a^	567.5^a^	617.9
SEM		3.5	8.3	7.5	12.9	14.6	24.6	18.2	50.7
Treatment (*n* = 3)									
SGC	96% AME_n_	24.3	72.3	95.1	185.9	284.2	429.4	452.2	595.6
	100% AME_n_	29.8	75.4	89.0	197.5	330.8	484.7	348.8	453.3
	104% AME_n_	31.3	91.5	105.9	222.6	326.1	507.1	447.4	573.2
	108% AME_n_	41.2	104.5	134.8	235.6	344.3	519.4	414.9	505.3
EGC	96% AME_n_	29.8	98.9	141.1	246.6	442.2	628.6	757.8	677.6
	100% AME_n_	37.9	99.7	158.2	253.5	477.2	747.4	666.3	616.0
	104% AME_n_	45.5	99.2	143.6	269.3	501.8	807.5	681.5	659.1
	108% AME_n_	52.5	134.8	171.1	319.0	572.0	782.5	720.0	730.5
	SEM	4.9	11.7	10.6	18.3	20.7	34.8	25.7	71.7
*P* value									
Growth curve (GC)		0.02	0.02	<0.001	<0.001	<0.001	<0.001	<0.001	0.02
Diet (factorial)		0.009	0.05	0.03	0.03	0.004	0.009	0.02	0.52
Diet (linear)		<0.001	0.007	0.007	0.002	<0.001	0.001	0.58	0.92
Diet (quadratic)		0.80	0.18	0.16	0.38	0.91	0.06	0.04	0.33
GC x Diet (factorial)		0.83	0.78	0.42	0.78	0.26	0.56	0.39	0.72
GC x Diet (linear)		0.44	0.94	0.39	0.60	0.08	0.29	0.67	0.45
GC x Diet (quadratic)		0.68	0.45	0.44	0.41	0.28	0.30	0.51	0.78

a–c LSmeans within a column and factor lacking a common superscript differ (*P* ≤ 0.05).

A linear relationship was observed between BW and body protein mass in growing broiler breeders (0–36 wk of age; Figure 1; *P* < 0.001). Separate lines had the best fit for each GC and each dietary energy-to-protein ratio. For SGC, the predicted body protein mass was expressed as −8.7+0.187*BW, whereas for EGC the predicted body protein mass was expressed as −5.6+0.182*BW (Figure 1A; *R*^2^ = 0.99; *P* < 0.001). For dietary energy-to-protein ratio, the constant coefficients (a) were estimated as −5.8, −5.9, −6.9, −7.3 and the linear coefficients (b) were estimated as 0.187, 0.184, 0.184, and 0.181 for 96, 100, 104, and 108% AME_n_ diet, respectively (Figure 1B; *R*^2^ = 0.99; *P* < 0.001). Although separate regression lines significantly improved the model fit for each GC and each dietary energy-to-protein ratio, absolute differences in predicted body protein mass at each given BW were small. Consequently, the common linear regression line is presented. A common linear regression line in growing breeders was expressed as −6.4+0.184*BW (*R*^2^ = 0.99; *P* < 0.001). In mature breeders (36–60 wk of age), a common line had the best fit for GC and dietary energy-to-protein ratio (126.4+0.15*BW; *R*^2^ = 0.86; *P* < 0.001) to predict body protein mass.

An exponential relationship was observed between BW and body fat mass in growing broiler breeders (0–36 wk of age; Figure 2; *P* < 0.001). A common line for both GC had the best fit for predicted body fat mass, which can be expressed as $-42.2+50.8*1.0006^{BW}\;$(Figure 2A; *R*^2^ = 0.98; *P* < 0.001). Separate lines had the best fit for each dietary energy-to-protein ratio, which was estimated with the following coefficients; the constant coefficients (a) were estimated as −31.5, −43.5, 38.6, and −74.7, the linear coefficients (b) were estimated as 39.6, 49.4, 47.5, and 82.5, and the exponential coefficients (c) were estimated as 1.0007, 1.0006, 1.0007, and 1.0005 for 96, 100, 104, and 108% AME_n_ diet, respectively (Figure 2B; *R*^2^ = 0.98; *P* = 0.03). In mature breeders (36–60 wk of age), a linear common line had the best fit for each GC and dietary energy-to-protein ratio (−811+0.35*BW; *R*^2^ = 0.61; *P* < 0.001) to predict body fat mass.

**Figure 3:**
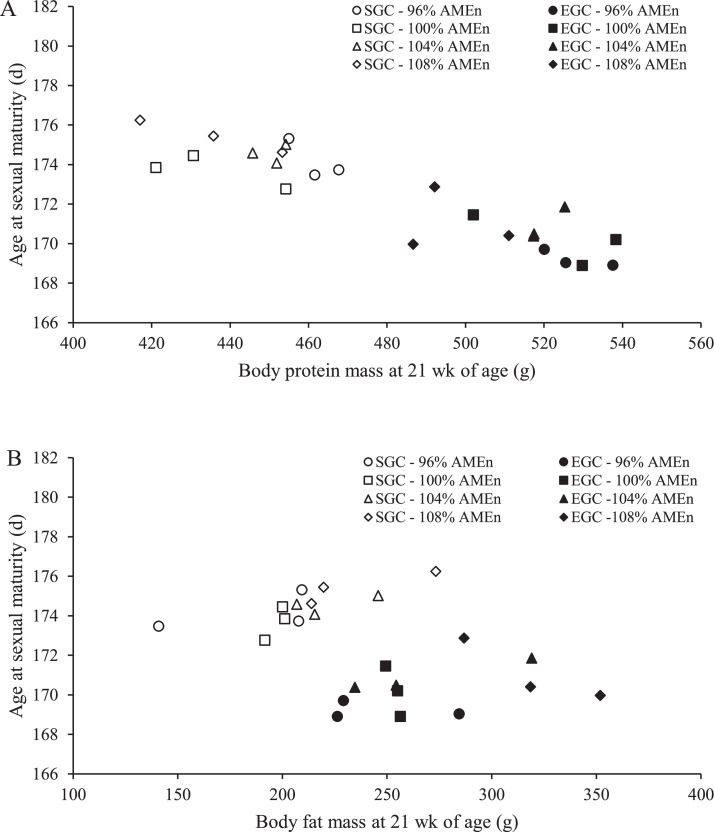
Relationship between body protein (A) and body fat (B) mass (g) at 21 wk of age and age at sexual maturity (age at 50% egg production; d) of broiler breeders fed at 2 different growth curves (SGC, standard growth curve or EGC, elevated growth curve (+15%) and 4 diets, differing in energy-to-protein ratio (96, 100, 104, or 108% AME_n_) from d 0 onward.. Each symbol represents 1 replicate (*n* = 24 pens).

ASM was related to body protein mass at 21 wk of age (Figure 3A; *R*^2^ = 0.83; *P* < 0.001). For each 100 g of body protein mass extra at 21 wk of age, ASM advanced with 5.4 d. The linear relationship was also observed at 6, 12 and 16 wk of age (*R*^2^ = 0.78, 0.71, and 0.78, respectively, all *P* < 0.001; data not shown). Body fat mass at 21 wk of age did not relate to ASM (Figure 3B; *R*^2^ = 0.19; *P* = 0.85), neither at other ages during rearing (*P* = 0.57, 0.39, and 0.69 for 6, 12, and 16 wk of age, respectively; data not shown). Body protein percentage and body fat percentage at 21 wk of age did not relate to ASM (*P* = 0.19 and 0.25, respectively, data not shown).

### Energetic Efficiency

**Figure 4:**
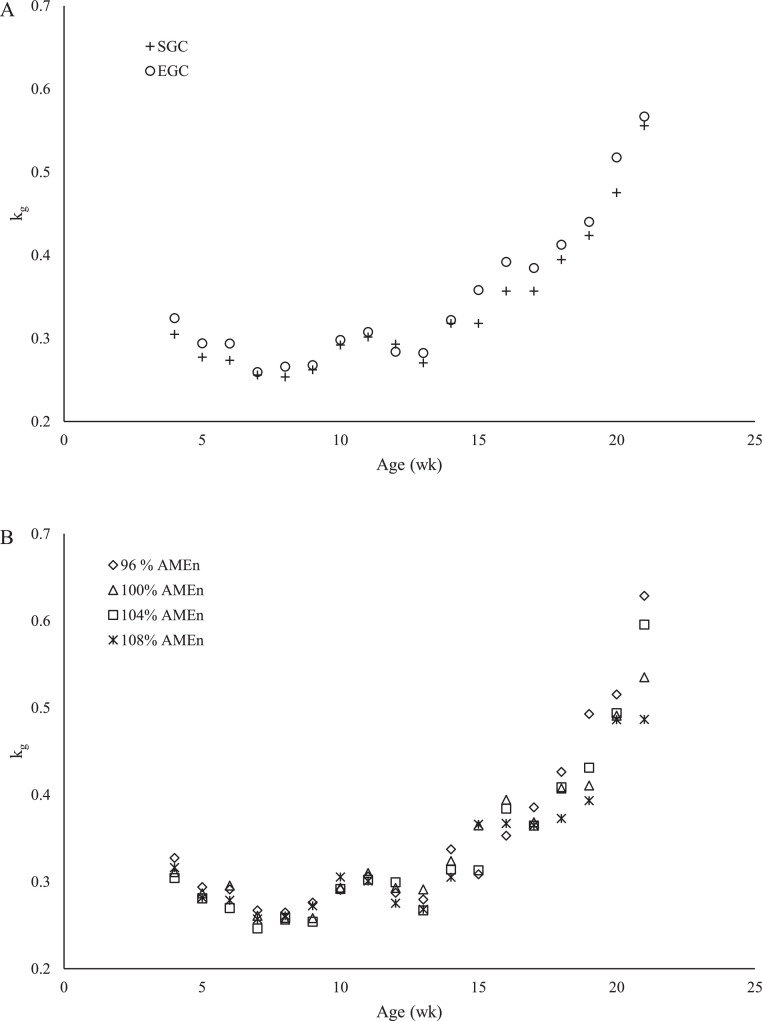
Relationship between broiler breeder age and calculated efficiency of energy utilization for body weight gain (k_g_) of broiler breeders between 0 and 21 wk of age fed at 2 different growth curves (A; SGC, standard growth curve or EGC, elevated growth curve (+15%)) and 4 diets (B), differing in energy-to-protein ratio (96, 100, 104, or 108% AME_n_) from d 0 onward. Each symbol represents the average calculated k_g_ per treatment at each time point.

Figure 4 presents the average calculated values for k_g_ for each GC (Figure 4A) and each dietary energy-to-protein ratio (Figure 4B) from 0 to 21 wk of age. A quadratic relationship between k_g_ and age was observed (*R*^2^ = 0.72; *P* < 0.001). Inclusion of GC and dietary energy-to-protein ratio further improved the model fit. Within SGC, parallel regression curves showed the best fit for each dietary energy-to-protein ratio, which can be expressed as $a-0.0304*Age+0.00173*Age^{2}$, where a were estimated as 0.418, 0.397, 0.386, and 0.381 for 96, 100, 104, and 108% AME_n_ diet, respectively (*R*^2^ = 0.74; *P* < 0.001). Within EGC, a common regression curve showed the best fit for all dietary energy-to-protein ratios, which can be expressed as $0.420-0.0334*Age+0.00189*Age^{2}$ (*R*^2^ = 0.73; *P* < 0.001). At 21 wk of age, average calculated k_g_ was 0.54, which was used for further calculations of k_e_ during the production period.

**Figure 5:**
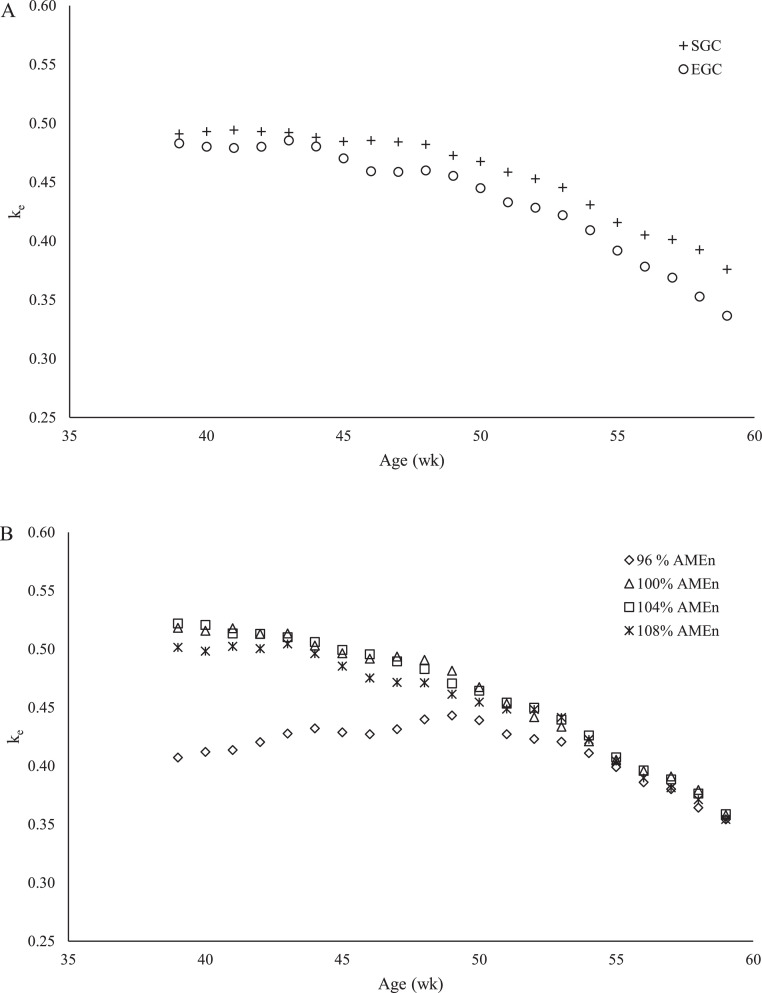
Relationship between broiler breeder age and calculated efficiency of energy utilization for egg production (k_e_) of broiler breeders between 36 and 60 wk of age fed at 2 different growth curves (A; SGC, standard growth curve or EGC, elevated growth curve (+15%)) and 4 diets (B), differing in energy-to-protein ratio (96, 100, 104, or 108% AME_n_) from d 0 onward. Each symbol represents the average calculated k_e_ per treatments at each time point.

Figure 5 presents the average calculated values for k_e_ for each GC (Figure 5A) and each dietary energy-to-protein ratio (Figure 5B) from 36 to 60 wk of age. A quadratic relationship between k_e_ and age was observed (*R*^2^ = 0.46; *P* < 0.001). Inclusion of GC and dietary energy-to-protein ratio further improved the model fit. Within SGC, parallel regression curves showed the best fit for each dietary energy-to-protein ratio, which can be expressed as $a+0.033*Age-0.00040*Age^{2}$, where a were estimated as −0.211, −0.186, −0.182, and −0.192 for 96, 100, 104, and 108% AME_n_ diet, respectively (*R*^2^ = 0.55; *P* = 0.001). Within EGC, separate lines had the best fit for each dietary energy-to-protein ratio, which was estimated with the following coefficients; the constant coefficients (a) were estimated as −1.552, 0.142, 0.463, and 0.043, the linear coefficients (b) were estimated as 0.081, 0.024, 0.010, and 0.026, and the quadratic coefficients (c) were estimated as −0.00082, −0.00034, −0.00021, and −0.00036 for 96, 100, 104, and 108% AME_n_ diet, respectively (*R*^2^ = 0.81; *P* < 0.001).

## DISCUSSION

### Dynamics in Body Composition

To our knowledge, the dynamics in body composition in broiler breeder hens from hatch till the end of the production period has not been investigated before. Other studies have only considered body composition during the rearing period (Sakomura et al., 2003; De Los Mozos et al., 2017), during sexual maturation (Rabello et al., 2006; Hadinia et al., 2020), or during the production period (Caldas et al., 2018; Salas et al., 2019), or only measured representatives of body composition, like abdominal fat pad and breast muscle weight (Van Emous et al., 2013; Zuidhof, 2018). Measuring body composition both during the rearing and production period allowed to model relationships between BW of the breeders and body composition in both growing breeder pullets and mature breeders. The models provided a means of calculating body composition according to variations in BW. For the models, fresh BW was used instead of feather-free BW. Defeathering of the carcass is required to obtain a homogeneous mixture for BC analysis. For practical applicability of the BC models though fresh BW was used, because a high correlation (*R*^2^ = 1.00) was observed between fresh BW and feather-free BW. Additionally, fresh BW is easy to measure in practice, whereas feather-free BW requires euthanization of the breeder and no differences between treatments were observed in feather weight as percentage of fresh BW (Heijmans et al., 2021).

Body protein mass is tightly regulated and mainly depended on BW of the breeder hen and to a lower extent on GC or dietary energy-to-protein ratio. Growing animals always have a basic daily body protein retention that they need to fulfill before additional body protein and fat can be retained (Boekholt et al., 1994; Boekholt and Schreurs, 1997). Sakomura et al. (2003) observed a comparable allometric relationship as presented in the current study between BW and body protein mass in growing breeder pullets of −9.1+0.171*BW. Predicted body protein mass was lower in the study of Sakomura et al. (2003), most probably due to differences in genetics (Hubbard Hi-Yield vs. Ross 308 breeders). When looking at body protein content, instead of body protein mass, other studies also observed a lack of difference in body protein percentage or breast muscle percentage when breeders were 8 to 20% heavier, compared to a standard BW according to breeder guidelines (Renema et al., 2001; Van Emous et al., 2013; Salas et al., 2019). This again indicates a tight regulation of body protein content. In the current study, at the same BW, a breeder on the EGC had a lower body protein mass, compared to a breeder on the SGC. This indicates that slower growth results in a higher protein content, although predicted differences were small, for example, ∆ = 7 g body protein mass at 2,000 g BW (∆ = 0.4%). A lower dietary energy-to-protein ratio resulted in a higher body protein mass, at the same BW, although predicted differences were again small, for example, ∆_max_ = 14 g body protein mass at 2,000 g BW (∆_max_ = 0.7%). This is in line with other studies, who observed a higher breast muscle weight, as representative for total body protein mass, when breeders were fed a diet with a lower dietary energy-to-protein ratio (Van Emous et al., 2013; Lesuisse et al., 2017). Feeding breeders a lower dietary energy-to-protein ratio, while aiming for a similar BW, resulted in a 5.4 to 22.8% higher dietary crude protein intake (Van Emous et al., 2013; Lesuisse et al., 2017; Heijmans et al., 2021). The surplus of dietary crude protein was thus only partly retained as (additional) body protein. In mature breeders, little further body protein growth occurred, due to a restriction in feed allowance and growth, as recommended by the breeder company (Aviagen, 2016b). This has also been observed by others (Nonis and Gous, 2016). It can be speculated that body protein growth will continue when breeders are allowed to grow further when feed allowance is further increased or when feed is provided ad libitum, as breeders have not reached their somatically mature weight yet (Gous, 2015; Zukiwsky et al., 2021).

Body fat mass showed an exponential relationship to BW. Sakomura et al. (2003) described a linear relationship between body fat mass and BW in growing breeders pullets of 7.0+0.085*BW. In that study, they only analyzed breeders up to approximately 2,000 g of BW, whereas the current study also included breeders up to 4,400 g of BW. When average weekly fat growth was calculated in growing breeders, based on Table 3, a fat growth spurt is observed after 16 wk of age (8.0 g/wk vs. 24.2 g/wk, 0–16 wk of age vs. 16–36 wk of age, respectively). This may explain why Sakomura et al. (2003) did not observe an exponential relationship, as the fat growth spurt occurred after approximately 2,000 g of BW. If we only analyzed data of breeders up to 2,000 g of BW, a linear regression curve showed a similar fit (*R*^2^ = 0.85 and BIC = 1,135) as an exponential regression curve (*R*^2^ = 0.85 and BIC = 1,136). A fat growth spurt toward the end of rearing was observed as well in layers (Kwakkel et al., 1993). It was speculated that the first fat growth is mainly deposited as intermuscular fat and the second fat growth spurt mainly as abdominal fat (Kwakkel et al., 1993). When calculating the proportion of abdominal fat to total fat, indeed we observe an increase from 6.5% at 16 wk of age to 13.2% at 36 wk of age. This indicates a faster accretion of abdominal fat at later ages, compared to nonabdominal fat in the body.

Body fat mass was higher in EGC breeders compared to SGC breeders at each age when body composition was determined. Other studies also observed a higher fat mass when breeders were 8 to 20% heavier, compared to a standard BW according to breeder guidelines (Renema et al., 2001; Sun and Coon, 2005; Van Emous et al., 2013; Salas et al., 2019). In the indicated studies, contrasts in GC were only maintained until 21 wk of age, resulting in breeders having a similar body fat mass during production, irrespective of initial BW and body fat mass differences at 21 wk of age (Renema et al., 2001; Sun and Coon, 2005; Van Emous et al., 2013; Salas et al., 2019). This is confirmed in the current study, as breeders had a similar predicted fat mass at the same BW, irrespective of GC. This indicates that body fat mass is related to BW rather than to growth rate.

Dietary treatments also had an effect on body fat mass. An increase in dietary energy-to-protein ratio resulted in a higher body fat mass at the same BW, that is, ∆_max_ = 45 g body fat mass at 2,000 g BW (∆_max_ = 2.3%). This is in line with other studies (Van Emous et al., 2013, 2015; Lesuisse et al., 2017, 2018; Salas et al., 2019). If a surplus of energy is supplied, this is mostly retained as fat (Boekholt et al., 1994; Leeson et al., 1996; Boekholt and Schreurs, 1997). It remains unclear whether or not differences in fat mass persist when contrasts in dietary treatments disappear. Van Emous et al. (2013) showed that breeders had more abdominal fat and thus more fat mass at 20 wk of age, when dietary energy-to-protein ratio increased by decreasing the dietary protein content. When breeders were fed a standard diet hereafter, a similar body fat mass was observed at 40 wk of age. It can thus be suggested that differences in dietary treatments should be maintained to maintain differences in body fat mass.

In mature breeders, after 36 wk of age, body fat mass decreased for all dietary treatments, except for the 96% AME_n_. Salas et al. (2019) also observed a decrease in fat mass after peak production. Two potential mechanisms might be involved in the decrease in fat mass; 1) body fat is mobilized to support yolk fat (Salas et al., 2017) or egg (Nonis and Gous, 2012) production or 2) body fat is mobilized to fulfill energy requirements for basic daily protein retention (Boekholt and Schreurs, 1997) as breeders have not reached their somatically mature weight yet (Gous, 2015; Zukiwsky et al., 2021). Breeders fed the 96% AME_n_ diet required a relative high feed intake to achieve pair-gaining (Heijmans et al., 2021), where the surplus of nutrients were deposited as fat. This indicates that these breeders were inefficient with their nutrients as mature breeders, which will be discussed further in the “energetic efficiency” paragraph below.

### Age at Sexual Maturity

Sexual maturation of breeders pullets is a complex process which depends on multiple factors (Hanlon et al., 2020). Several authors emphasized the importance of metabolic status on sexual maturation (Bédécarrats et al., 2016; Hanlon et al., 2020; Van der Klein et al., 2020). Discrepancy exists whether a body protein (Sun et al., 2006; Eitan et al., 2014; Salas et al., 2019) or body fat (Zuidhof, 2018; Van der Klein et al., 2018b; Hadinia et al., 2020) threshold exists for sexual maturation, although none of the studies indicated above correlated body composition directly to sexual maturation. The current study shows a clear relationship between body protein mass at a given age during rearing and sexual maturation, where each 100 g extra body protein mass advanced sexual maturation with 5.4 d. In line with this, Lewis et al. (2007) observed that with each 100 g extra BW at 20 wk of age, sexual maturation advanced with 2 d. These results indicate that particularly body protein mass is important for sexual maturation. Two potential mechanisms might be involved. First, protein is an important component of the oviduct and ovary (Ricklefs, 1976; Bowmaker and Gous, 1989; Kwakkel et al., 1993). An advanced development of the reproductive tract might have led to a higher total body protein mass. Future studies to sexual maturation should therefore include growth and composition of the reproductive tract in breeder pullets. Second, body protein is an important source for yolk protein (Ekmay et al., 2014) and yolk fat, via gluconeogenesis (Boonsinchai, 2015) and de novo lipogenesis (Salas et al., 2017) in young breeders. Around sexual maturation, an increase in body protein mobilization is observed (Vignale et al., 2017, 2018), indicating breeders use body protein reserves to support egg production. Body fat mass was not related to sexual maturation in the current study. This indicates either that body fat mass does not play a role in sexual maturation or that it was already beyond the threshold needed for sexual maturation. In the studies that hypothesized that body fat plays an important role in sexual maturation, results were either confounded with BW (Hadinia et al., 2020), and thus body protein, or body composition was measured in laying and nonlaying breeders at 52 (Zuidhof, 2018) or 55 (Van der Klein et al., 2018b) wk of age and not around sexual maturation.

### Dynamics in Energetic Efficiency

To our knowledge, no other studies are available that attempt to model k_g_ and k_e_ in relation to age of the breeders. Quantifying factors that contribute to energy efficiency is challenging, but this can have profound economic and environmental consequences (Zuidhof, 2019). For the calculations of maintenance requirement only body protein mass was taken into account, as this was assumed as the metabolic active component of the body (Emmans, 1987; Gous, 2015; Nonis and Gous, 2018). Body fat is considered as inert and therefore does not require maintenance (Emmans, 1987; Gous, 2015; Nonis and Gous, 2018). One could argue that fatter breeders with a similar body protein mass as leaner breeders have a higher maintenance requirement as they have to carry more weight. Therefore, calculations were also performed using a ME_m_ formula which takes BW instead of body protein into account (Noblet et al., 2015). Absolute values for k_g_ were on average 0.11 higher and absolute values for k_e_ were on average 0.04 lower with that ME_m_ formula. The shape of the regression curves (quadratic relationship) and the treatment effects remained the same as with the body protein maintenance formula.

The current study shows a quadratic relationship between k_g_ and age of the pullets. Values for k_g_ ranged from 0.27 (8.8 wk of age) to 0.54 (21 wk of age). The calculated value of k_g_ at 21 wk of age (0.54) is comparable to reported k_g_ values of breeders during production (Rabello et al., 2006; Reyes et al., 2011, 2012). Rabello et al. (2006) calculated a k_g_ of 0.47 in Hubbard Hi-Yield breeders between 26 and 33 wk of age. Reyes et al. (2011, 2012) calculated a k_g_ of 0.59 in Cobb 500 breeders between 32 and 42 wk of age and 0.57 between 53 and 62 wk of age. The calculated values of k_g_ during rearing are lower compared to k_g_ values reported by Sakomura et al. (2003). They observed values for k_g_ of 0.79 (3–8 wk of age), 0.64 (9–14 wk of age), and 0.81 (15–20 wk of age) in Hubbard Hi-Yield breeders (Sakomura et al., 2003). The values presented in literature vary substantially due to differences in animal factors (e.g., age, genetic strain), environmental factors (e.g., ambient temperature), dietary factors (e.g., chemical composition of the diet) (Zuidhof, 2019), and methodologies used for determination of energetic efficiency (Sakomura et al., 2003).

Even though absolute values of k_g_ during rearing were higher in Sakomura et al. (2003), they also observed a quadratic shape for k_g_ during rearing. The shape of the quadratic regression line for k_g_ might be explained by feed restriction levels. Feed restriction is most severe between 7 and 16 wk of age (25–33% of ad libitum), whereas this is less severe during the production period (50–90% of ad libitum) (De Jong and Guémené, 2011). It can be hypothesized that a more severe feed restriction between 7 and 16 wk of age results in a lower energetic efficiency, compared to ages outside this range. Pullets might mobilize body fat during periods of severe feed restriction, resulting in a higher heat production and thus lower efficiency, in order to meet their energy requirements for basic daily body protein retention (Boekholt et al., 1994; Boekholt and Schreurs, 1997). In line with this hypothesis, within SGC pullets, a higher dietary energy-to-protein ratio resulted in a lower predicted k_g_. An increase in dietary energy-to-protein ratio resulted in a lower feed allowance to obtain pair-gaining (Heijmans et al., 2021) and thus a more severe feed restriction, although differences in k_g_ between dietary treatments were relatively small (∆_max_ = 0.04). The dietary effect on k_g_ was not observed within EGC pullets. For EGC pullets, predicted values of k_g_ were even lower than predicted values of k_g_ for SGC pullets on the 96% AME_n_ diet, whereas EGC pullets had a higher feed allowance (Heijmans et al., 2021). It remains unclear why dietary energy-to-protein ratio did not affect k_g_ in EGC pullets. Future studies should investigate energetic efficiency for breeder pullets in restricted and ad libitum fed pullets to confirm the impact of feed restriction level on energetic efficiency.

A quadratic relationship was also observed between k_e_ and age of the breeders. Predicted values of k_e_ ranged from 0.28 to 0.56 between 36 and 60 wk of age. For the calculations, in case average BW gain was negative, a growth of zero was assumed (3 pens; −1.1, −2.1, and −5.2 g/d average BW gain), as it remains unclear whether or not a negative BW gain yields energy or if there is a cost factor involved as well. If calculations were performed assuming a negative average BW gain only yields energy, average values of k_e_ were 0.001 lower. The shape of the regression curve and the treatment effects remained the same. The predicted values of k_e_ are lower compared to calculated k_e_ values in other studies with breeders. Rabello et al. (2006) calculated a k_e_ of 0.64 in Hubbard Hi-Yield breeders between 26 and 33 wk of age. Reyes et al. (2011, 2012) calculated a k_e_ of 0.73 in Cobb 500 breeders between 32 and 42 wk of age and 0.66 between 53 and 62 wk of age. Again, differences in k_e_ values might be due to differences in animal factors, environmental factors, dietary factors and methodologies used for calculations (Sakomura et al., 2003; Zuidhof, 2019), where the latter one potentially has the largest effect on differences in k_e_ values.

Predicted k_e_ decreased with 0.13 on average with age of the breeders. The decrease of k_e_ with age might partly be explained by a decrease in feather cover with increasing breeder age (Heijmans et al., 2021). Lower feather coverage will result in a higher maintenance requirement, as feathers provide insulation to the hen (Van Krimpen et al., 2014). Van Krimpen et al. (2014) calculated in laying hens that with each percent of feather coverage loss, this will require 0.23 kcal/d extra. In the current study, feather cover was 100% at 21 wk of age and decreased to approximately 68% at 59 wk of age (*P* < 0.001; Heijmans, unpublished data). This corresponds to max 7.4 kcal/d extra to correct for feather coverage. If feather coverage was taken into account for k_e_ calculations, average k_e_ values were 0.01 higher from 36 to 46 wk of age and 0.02 higher from 46 wk of age onward, compared to k_e_ values when feather coverage was not taken into account. Hence, feather coverage did not explain the decrease of k_e_ with age. The decrease in k_e_ with age of the breeders is probably mostly attributed to a decrease in laying rate, as ME_int_, ME_m_, and ME_g_ were quite constant from 36 to 60 wk of age and egg weight increased with age (Heijmans et al., 2022). It can thus be speculated that strategies aiming for a more persistent laying rate will also improve energetic efficiency.

For SGC breeders, k_e_ was 0.02 higher on average, compared to EGC breeders. This indicates that SGC breeders relatively retain more energy in eggs than EGC breeders. Both GC were fed a restricted amount of feed, but EGC breeders had a 15% higher feed allowance during production, compared to SGC breeders (Heijmans et al., 2021). It can be speculated that EGC breeders had less fasting time during the day, compared to SGC breeders. Fasting can improve digestibility of metabolizable energy in the diet with 1.8% compared to nonfasting (Wang et al., 2022). Assuming a 1.8% higher AME_n_ availability for SGC breeders (on average 7.7 kcal/d), would result in a 0.02 lower predicted k_e_ value on average for SGC breeders, which is then comparable to predicted k_e_ values for EGC breeders. Dietary energy-to-protein ratio had minimal effects on predicted k_e_ values, with exception of the 96% AME_n_ dietary treatment in EGC breeders. Up to approximately 50 wk of age, EGC breeder fed the 96% AME_n_ diet had a remarkably lower predicted k_e_ values (up to 0.27 lower), compared to the other dietary EGC treatments. These breeders required a high feed allowance for pair-gaining from approximately 32 to 50 wk of age (Heijmans et al., 2021), whereas this only resulted in a slight increase in egg weight and did not affect laying rate (Heijmans et al., 2022), compared to the other EGC dietary treatments. Although eating time was not determined in the current study, visually it was observed that these breeders were fed close to ad libitum (10–12 h feed availability). Potential heat producing activities, related to high feed intake, were not taken into account in the calculations. It can be speculated that predicted k_e_ values of 96% AME_n_ EGC breeders will be closer to the predicted k_e_ values of other dietary treatments if the energy consuming activities, like longer eating and more digestive processes, were taken into account.

## CONCLUSIONS

It can be concluded that a linear relationship exists between body protein and BW of the breeder hen, with minimal effects of dietary treatments. Body protein is one of the factors determining sexual maturation in breeder pullets. Body fat mass showed an exponential relationship to BW, with a fat growth spurt toward the end of rearing and start of production. An increase in dietary energy-to-protein ratio results in a higher body fat mass, at the same BW. Dietary treatments had minimal effects on estimated energetic efficiency in breeders, whereas age had a pronounced effect. Energetic efficiency for BW gain was lower in pullets from 7 to 16 wk of age, compared to younger or older breeder pullets. Energetic efficiency for egg production decreased with age of the breeders, which was mostly related to a lower laying rate.

## Appendix Supplementary materials

Image, application 1
