# Supplementary material for: Changes in body composition and energetic efficiency in response to growth curve and dietary energy-to-protein ratio in broiler breeders
Source: Poult Sci. 2022 Dec 9;102(2):102410. doi: 10.1016/j.psj.2022.102410 (PMC9801220; doi:10.1016/j.psj.2022.102410)
Supplement: Supplementary file 1 [file mmc1.docx]

# SUPPLEMENTARY INFORMATION

**Table S1.** Defeathered body weight of broiler breeders selected for body composition analysis from 6 to 60 wk of age with 2 different growth curves (SGC = standard growth curve or EGC = elevated growth curve (+15%)) and 4 diets, differing in energy-to-protein ratio (96, 100, 104, or 108% AME_n_), fed from 0 to 60 wk of age.

|  |  |  | Age (wk)^1^ | | | | | | | | |
| --- | --- | --- | --- | --- | --- | --- | --- | --- | --- | --- | --- |
| Item | |  | 6 | 12 | 16 | 21 | 28 | 36 | 46 | 60 |  |
| Growth curve (n=12) | | |  |  |  |  |  |  |  |  |  |
|  | SGC |  | 686^b^ | 1296^b^ | 1771^b^ | 2327^b^ | 3072^b^ | 3525 | 3569 | 3745^b^ |  |
|  | EGC |  | 796^a^ | 1485^a^ | 2036^a^ | 2680^a^ | 3623^a^ | 4225 | 4195 | 4343^a^ |  |
|  | SEM |  | 5 | 8 | 13 | 7 | 11 | 6 | 15 | 16 |  |
| Diet (n=6) | |  |  |  |  |  |  |  |  |  |  |
|  | 96% AME_n_ | | 743 | 1394 | 1912 | 2502 | 3333 | 3784 | 3943 | 4046 |  |
|  | 100% AME_n_ | | 739 | 1383 | 1896 | 2516 | 3345 | 3895 | 3885 | 4030 |  |
|  | 104% AME_n_ | | 741 | 1387 | 1902 | 2513 | 3334 | 3911 | 3872 | 4033 |  |
|  | 108% AME_n_ | | 741 | 1399 | 1904 | 2483 | 3379 | 3910 | 3827 | 4066 |  |
|  | SEM |  | 7 | 12 | 18 | 11 | 15 | 8 | 21 | 23 |  |
| Treatment (n=3) | | |  |  |  |  |  |  |  |  |  |
|  | SGC | 96% AME_n_ | 686 | 1299 | 1787 | 2328 | 3050 | 3510^d^ | 3553^cd^ | 3741 |  |
|  |  | 100% AME_n_ | 687 | 1291 | 1755 | 2356 | 3076 | 3532^d^ | 3615^c^ | 3707 |  |
|  |  | 104% AME_n_ | 683 | 1297 | 1777 | 2332 | 3063 | 3525^d^ | 3589^cd^ | 3749 |  |
|  |  | 108% AME_n_ | 690 | 1298 | 1766 | 2292 | 3099 | 3531^d^ | 3520^d^ | 3783 |  |
|  | EGC | 96% AME_n_ | 801 | 1489 | 2037 | 2676 | 3616 | 4057^c^ | 4333^a^ | 4351 |  |
|  |  | 100% AME_n_ | 791 | 1474 | 2036 | 2677 | 3613 | 4259^b^ | 4156^b^ | 4353 |  |
|  |  | 104% AME_n_ | 799 | 1476 | 2026 | 2694 | 3605 | 4296^a^ | 4155^b^ | 4316 |  |
|  |  | 108% AME_n_ | 792 | 1500 | 2043 | 2673 | 3659 | 4289^ab^ | 4135^b^ | 4350 |  |
|  |  | SEM | 10 | 16 | 25 | 15 | 21 | 11 | 30 | 33 |  |
| P-value | |  |  |  |  |  |  |  |  |  |  |
|  | Growth curve (GC) | | <0.001 | <0.001 | <0.001 | <0.001 | <0.001 | <0.001 | <0.001 | <0.001 |  |
|  | Diet (factorial) | | 0.99 | 0.76 | 0.94 | 0.15 | 0.15 | <0.001 | 0.02 | 0.69 |  |
|  | Diet (linear) | | 0.88 | 0.72 | 0.84 | 0.20 | 0.07 | <0.001 | 0.001 | 0.54 |  |
|  | Diet (quadratic) | | 0.75 | 0.30 | 0.59 | 0.05 | 0.28 | <0.001 | 0.76 | 0.29 |  |
|  | GC x Diet (factorial) | | 0.87 | 0.91 | 0.87 | 0.28 | 0.88 | <0.001 | 0.005 | 0.59 |  |
|  | GC x Diet (linear) | | 0.71 | 0.74 | 0.75 | 0.15 | 0.94 | <0.001 | 0.03 | 0.32 |  |
|  | GC x Diet (quadratic) | | 0.92 | 0.52 | 0.99 | 0.29 | 0.43 | <0.001 | 0.004 | 0.69 |  |

*^a-d^LSmeans within a column and factor lacking a common superscript differ (P≤0.05).*

*^1^At 2 wk of age, defeathered BW was 232 g of the 96% AME_n_ diet and 264 g of the 108% AME_n_ diet.*
